# A comparison study of adaptive scale estimation in correlation filter-based visual tracking methods

**DOI:** 10.1186/s40638-017-0066-2

**Published:** 2017-11-02

**Authors:** Z. L. Wang, B. G. Cai

**Affiliations:** Beijing Jiaotong University, Beijing, 100044 China

## Abstract

Recently, discriminative correlation filter-based method becomes one of the popular directions in the field of visual tracking because of its computational efficiency and excellent performance, which make it especially suitable for real-time application. Most of them are focused only on the transition estimation. However, accurate scale estimation of the target plays a very important role in long-term tracking task and is still a challenging problem. The principle of CF-based visual tracking is introduced first. The approaches of adaptive scale estimation in correlation filter-based visual tracking methods are summarized in this paper, and their performances are analyzed by experiment comparison. The works here can provide a better understanding on the scale estimation problem for correlation filter-based visual tracking. Furthermore, maybe with the same strategy, other factors in visual tracking, such as appearance variation, can be integrated into the framework to improve the performance of correlation filter-based method.

## Introduction

Visual object tracking is one of the core problems of computer vision and used in a wide range of applications, such as intelligent human–computer interaction, security, video surveillance and analysis, compression, augmented reality, traffic control, medical imaging and video editing [1]. It also forms a basic part of higher-level vision tasks such as scene analysis and behavior recognition. Although visual object tracking has been studied for several decades and considerable progress has been made in recent years [2, 3], robust visual object tracking is still an open research problem in the field of computer vision, and there are some challenging factors for visual tracking, such as appearance changing, scale variations, occlusions, motion blur, fast motion, some factors caused by the motion between the object and camera and some others come from the environment, such as illumination change.

In view of its wide range of applications, considerable works in the field of object tracking have been done during the past few decades, and Ref. [4] makes an insightful review on this topic. Generally speaking, the existing tracking approaches can be classified into two groups according to the appearance model, discriminative model-based or generative model-based. Generative model-based trackers aim to build the metric model using, e.g., statistical models or templates to search the most similar patches for the tracked object [5–7]. On the other hand, discriminative model-based methods usually employ the binary classifier or machine learning techniques to distinguish the tracked object from the background. Some classifiers, such as support vector machine (SVM), structured output SVM [8], ranking SVM [9], boosting, semi-boosting and online multi-instance boosting [10], have been proposed for object tracking. SCM [11] even combines the discriminative classifier and generative model to achieve the high accuracy and robustness. But it involves with the heavy computational cost, which hinders its capability on real-time applications.

Recently, discriminative correlation filter (DCF) has successfully been applied to visual tracking [12–15] and the result performance is impressive, especially on its efficiency. As described in convolution theorem, the correlation in time domain corresponds to an element-wise multiplication in Fourier domain. Thus, the idea in nature of correlation filter is that the correlation can be calculated in Fourier domain in order to avoid the time-consuming convolution operation. Meanwhile, the correlation filter is treated as similarity measurement between the two signals in signal processing, which gives a reliable distance metric and explains the reason of the promising performance achieved by the proposed approaches. The correlation-based trackers learn a DCF for locating the target in each new frame. Some works are focused on the basic conceptions of correlation filter and exploiting the circulant structure. For example, Bolme et al. [15] trained the filter by minimizing the total squared error between the actual and the desired correlation output on a set of sample grayscale patches. By using circular correlation, the authors showed that the resulting filter can be computed efficiently using only FFTs and point-wise operations. Henriques et al. [16] further showed that the DCF formulation equivalently can be cast as learning a least squares regressor (ridge regression) on the set of all cyclic shifts of the involved training sample patches. This formulation was then used to introduce fast kernelized correlation filters. Some other basic works are conducted in [17].

Several works have recently addressed the generalization of a DCF tracker. Galoogahi et al. [18] extend the DCF with multi-channel filter and use HoG feature in CF. To improve the stability of correlation filter output, the color information is integrated in [12, 19]. However, because the multi-channel and multi-property of the target are employed in the CF, this kind of filter cannot directly apply to the online tracking problem. Alternatively, approximate formulations for learning multi-channel filters have been investigated for visual tracking [12, 13]. Danelljan et al. [12] introduced an adaptive feature dimensionality reduction technique to reduce the computational cost while preserving tracking performance.

Experiments with the benchmark dataset both in OTB [20] and VOT [21] show that DCF-based visual trackers present excellent performance, such as the capability of accurate target localization even in many different challenging tracking scenarios. Particularly, these trackers have the advantage of computing efficiency, which making them especially suitable for the real-time application. The significant gain in speed is obtained by exploiting the fast Fourier transform (FFT) at both learning and detection stages. However, most methods that employ DCFs for tracking are mainly restricted to translation estimation. This limits the performance of CF-based method; especially for long-term visual tracking, it is always the case in mobile robot and visual surveillance.

For long-term visual tracking task, many factors may affect the performance of the trackers, such as illumination variation, occlusion, scale changing and disappearance/reappearance, and the DCF-based tracker may imply poor performance under these situations. In this paper, we will focus on the scale estimation issue of DCF-based visual tracking. It is one of the most important factors in long-term visual tracking and our works also confirmed it. The nature for this is that, for the long-term visual tracking of discriminative model-based method, a big well-known issue is the stability–plasticity dilemma [22, 23]. That is, if we use some stable samples, such as the target assigned in the first frame, to train the classifier, then the tracker is unlikely to drifting and more robust to occlusions. However, if the target appearance variation is not taken into account in this case, the tracker is doomed to work not well in long-term visual tracking process. Furthermore, the capability of accurately retrieving the target scale is beneficial in many tracking applications. Here, we first make a short summary on the existing scale estimation method, and then, experiment comparisons among these methods have been conducted to get a deep insight on this issue. And some problems on the benchmark dataset are discussed.

## Scale estimation in DCF-based visual tracking

The importance of accurate scale estimation for visual tracking has been shown in many works, especially in Ref. [14, 24]. And several works addressed the scale estimation issues in CF-based tracking method. For example, Li et al. [25] proposed a kernelized correlation translation filter with multi-resolution extension. To solve the scale estimation problem, the target in different scales are sampled first and then resized these samples into a prefixed size, and the scale with the highest correlation score is regarded as the final result. However, to get sufficient scale accuracy, the translation filter needs to be run at several resolution layers, and this brings a higher computational cost. By incorporating context information into filter learning, Zhang et al. [17] estimate the scale variation based on consecutive correlation results. In DSST tracker [14], a HOG feature-based adaptive multi-scale correlation filter is learned to cope with the scale change problem. By learning the appearance changes caused by scale variations directly, and using fused features such as raw intensity value and HOG feature, DSST tracker can estimate the target scale adaptively and track at a higher frame rate. However, this method does not address the online model updating issue. And these correlation filter-based trackers are susceptible to drifting. Danelljan et al. [26] employed an adaptive feature dimensionality reduction method as in Ref. [27] to reduce the computational cost, while tracking the performance is preserved. A collaborative correlation tracker is proposed in [28]. The experiment results [14, 24] show that by combining the scale estimation with translation filter, their approaches outperform 19 state-of-the-art trackers in the OTB dataset.

In this section, we first briefly introduce the basic correlation filter-based visual tracking method; then, three typical strategies for scale estimation are presented, that is, multi-resolution-based [29], joint scale space filter, iterative joint scale space [14, 24].

### Basic correlation filter-based visual tracker

Correlation filters have been used in many applications such as object detection and recognition. Since the operator is readily transferred into the Fourier domain as element-wise multiplication, correlation filters have attracted considerable attention recently to visual tracking due to its computational efficiency. Bolme et al. [15] propose to learn a minimum output sum of squared error (MOSSE) filter for visual tracking on grayscale images, where the learned filter encodes target appearance with update on every frame. Many DCF-based visual tracking methods take it as a baseline approach. The basic idea of this method is as follows.

According to the convolution theorem, the correlation becomes an element-wise multiplication in the Fourier domain. To create a fast tracker, correlation is computed in the Fourier domain with fast Fourier transform (FFT). First, the 2D Fourier transform of the input image, *F* = *F*(*f*), and of the filter, *H* = *F*(*h*), is computed. We use the ⊙ ← symbol to explicitly denote element-wise multiplication and ∗ ← to indicate the complex conjugate; then, correlation takes the form:

1 $$G = F \odot H^{*}$$

The correlation output is transformed back into the spatial domain using the inverse FFT. The target location corresponds to the maximum value in the correlation output. Generally, a 2D Gaussian shape is expected for the correlation output, which peak is centered on the target in training image.

For the first frame, the filter is learned according to the provided input image and the expected correlation output, and it is 2D Gaussian output. Let us *f*_*i*_ is a set of training images and *g*_*i*_ is generated from the 2D Gaussian shape; then,

2 $$H_{i}^{*} = \frac{{G_{i} }}{{F_{i} }},$$

where the division is performed element-wise. To find a filter that maps training inputs to the desired training outputs, for the MOSSE method, a filter ***H*** is defined as that minimizes the sum of squared error between the actual output of the convolution and the desired output of the convolution. The cost function for this optimization problem is defined as

3 $$\mathop {\hbox{min} }\limits_{{H^{*} }} \sum\limits_{i} {\left| {F_{i} \odot H^{*} - G_{i} } \right|^{2} }$$

By solving for *H* ^*^, a closed form expression for the MOSSE filter is found:

4 $$H^{*} = \frac{{\sum\nolimits_{i} {G_{i} \odot F_{i}^{*} } }}{{\sum\nolimits_{i} {F_{i} \odot F_{i}^{*} } }}$$

In the following frames, an online update of *H* ^*^ is then performed based on that new location, such as

5 $$H^{*} = \eta \frac{{G_{i} \odot F_{i}^{*} }}{{F_{i} \odot F_{i}^{*} }} + (1 - \eta )H_{i - 1}^{*}$$

or in a more practical form as in MOSSE filter as

6 $$H_{i}^{*} = \frac{{A_{i} }}{{B_{i} }},$$

where

$$\begin{aligned} A_{i} & = \eta G_{i} \odot F_{i}^{*} + (1 - \eta )A_{i - 1} \\ B_{i} & = \eta F_{i} \odot F_{i}^{*} + (1 - \eta )B_{i - 1} \\ \end{aligned}$$

The computational complexity of DCF-based tracking is $O(N\log N)$, where *N* is the number of pixels in the filter. This comes from the FFTs used during the correlation operation and the online update. The advantages of DCF-based method are easy to implement and can be just accurate and much faster. Under the framework of DCF tracking, some works try to further improve its performance by taking multi-channel features, spatial constraints into consideration. But most of these works are restricted to translation estimate, and this implies poor performance when encounter with significant variations in the target scale.

### Multi-resolution-based scale estimation

For the object detection problem, a standard approach to eliminate the scale effect is to apply a detecting process at multiple resolutions. Accordingly, to tackle the problem of the fixed template size in correlation filter tracker, Li et al. [25] proposed an effective scale-adaptive scheme. Moreover, they integrate HoG and color-naming feature together to further boost the overall tracking performance. It is called SAMF (scale-adaptive multiple-feature) tracker. SAMF is the improvement in kernel-based correlation filter; to solve the scale change issue in object tracking, a sample searching strategy is implied. Here, only the scale estimation strategy in this method is briefly introduced.

Let the template size be $S_{T} = (s_{x} ,s_{y} )$, and *s*_*x*_ and *s*_*y*_ denote the horizontal and vertical size, respectively, and define a scaling pool as $S = \left\{ {t_{1} ,t_{2} , \ldots ,t_{k} } \right\}$. Every time, the target window size *s*_*t*_ in the original image space is resampled *k* sizes in $\{ t_{i} s_{t} |t_{i} \in S\}$. These samples are resized into the same size with fixed template *S*_*T*_, to match the requirement of the element-wise dot product in correlation filter. The final response is calculated by

7 $$\arg \hbox{max} \;F^{ - 1} \hat{f}(Z^{{t_{i} }} ),$$

where $z^{{t_{i} }}$ is the sample patch with the size of $t_{i} s_{t}$, which is resized to *S*_*T*_. Since the result of the response function is a vector, the max operation is employed to find its maximum scalar. As the target movement is implied in the response map, the final displacement needs to be tuned by *t* to get the real movement bias. The updating procedure is almost same as other DCF-based method.

In their experiments, the scale pool is set as:

$$S = \{ 0.985,0.99,0.995,1.0,1.005,1.01,1.015\} .$$

Though only seven different scale spaces are used, and all the parameters are same for the experiments, with the benchmark dataset of VOT2014, the results are impressive. One big difference to others is that the scale estimation is included in SAMF.

### Joint scale space estimation

The fused feature-based translation estimation and the scale estimation are separately processed in SAMF. The final results of translation estimation will be tuned to get a more accurate result. Instead of estimating the translation and scale separately, joint scale space-based method tries to jointly estimate the translation and scale of the target. It is achieved by computing the correlation scores in a box-shaped region of a scale pyramid representation. Both translation and scale estimates are then achieved by maximizing this score.

**Fig. 1:**
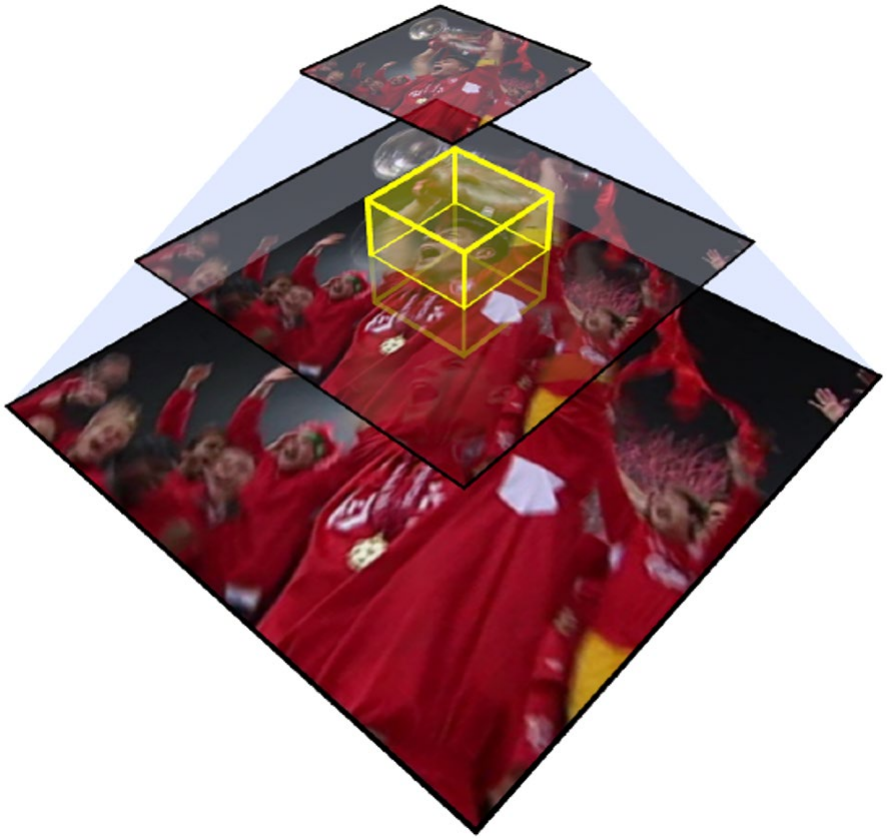
Architecture of joint scale space estimation method [24]

To update the joint scale space filter, a feature pyramid in a rectangular area around the given target location is first constructed. As shown in Fig. 1, the feature pyramid is constructed such that the target size at the current scale corresponds to the spatial filter dimensions *M* * *N*. The training sample *f*_t_ is set to the rectangular cuboid of size *M* * *N* * *S* centered around the target location and scale. Here, *S* corresponds to the number of the scale space. The joint scale space filter can be updated with formula  (6), using a three-dimensional Gaussian function as the desired correlation output *g*.

Obviously, joint scale space-based method suffers from the computational cost and is not suitable for real-time application. Another issue is because the feature pyramid at the detection step is constructed around the predicted target location. This might result in an inclusion of a shearing component in the transformation relating the test sample *z*_*t*_ to the feature pyramid constructed around the actual target center. The shearing effect is caused by errors in the predicted target location. This significantly affects the performance of the joint scale space filter by introducing a bias in the translation estimate.

### Iterative scale space estimation

To reduce the impact of the scale space shearing distortion, the iterative scale space filter strategy can be employed. In this method, given a new frame, first using the previous target location and scale for the filter to estimate the translation, generally, a standard translation filter is used. Then the scale estimation is something a little like multi-resolution method, which uses a search strategy in scale space, and the scale is correspondent to the maximum correlation score. This procedure is performed iteratively until the convergence is achieved.

Typically, based on the observation that the target scale variation between two frames is small compared to the change in translation, the translation filter *h*_t,*trans*_ is carried out first to get the new target location; then, scale filter *h*_*t*,scale_ is applied. The test sample for scale estimation *h*_*z*,scale_ is extracted from the new location. In many cases, the iterative step may be not necessary, just as in discriminative scale space tracking (DSST) method [24].

**Fig. 2:**
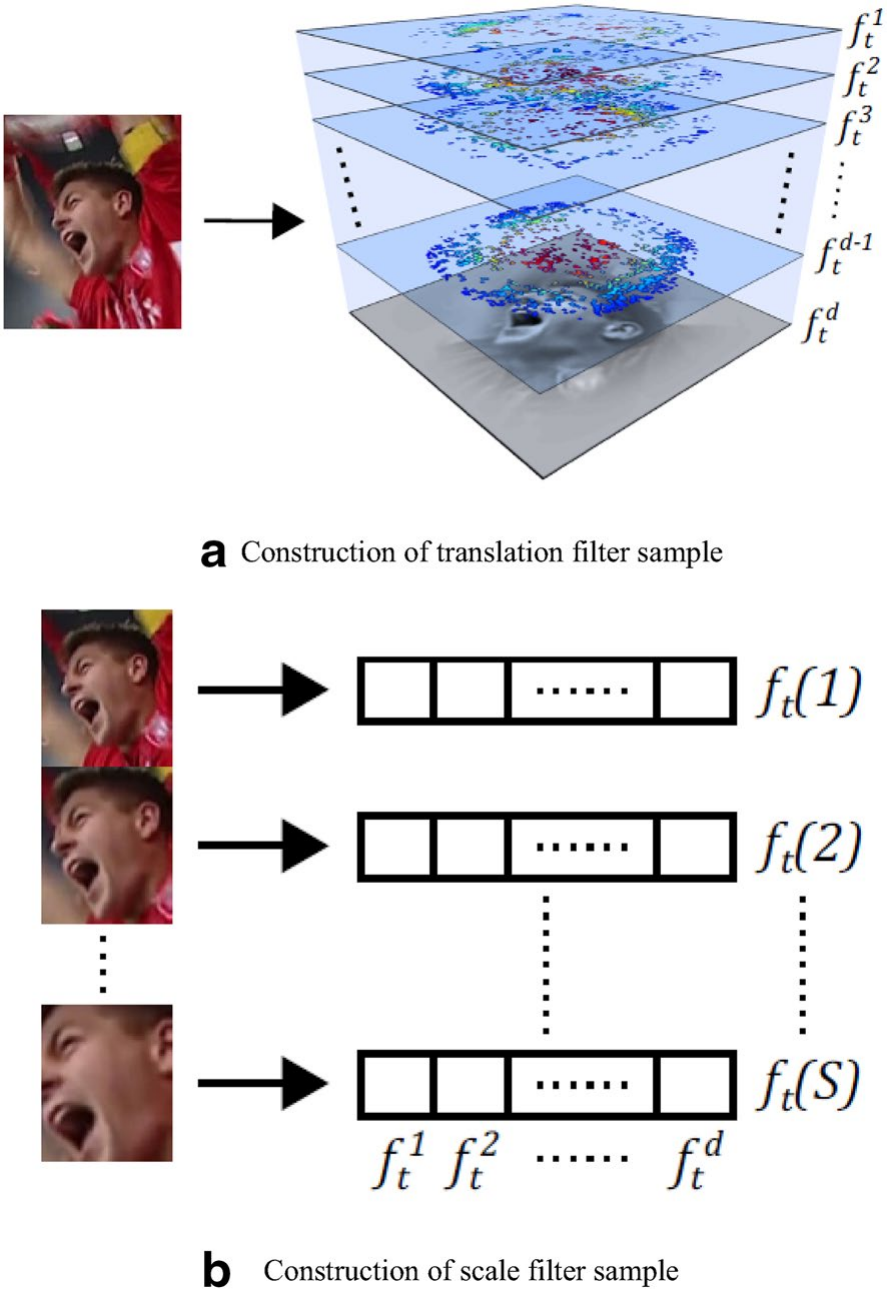
Training samples used in DSST and fDSST method. **a** Construction of translation filter sample. **b** Construction of scale filter sample

As shown in Fig. 2, the DSST method uses a 2D multi-channel features for translation filter and a separate 1D scale filter for scale estimation. To construct the training sample *f*_*t*,scale_, the features are extracted using variable patch sizes centered around the target. Let *P* × *R* denote the target size in the current frame and *S* the size of the scale filter. For each

$$n \in \left\{ {{\text{floor}} \left( -{\frac{S - 1}{2}} \right), \ldots ,{\text{floor}} \left( {\frac{S - 1}{2}} \right)} \right\},$$

an image patch *I*_*n*_ of size *a* ^*n*^ *P* × *a* ^*n*^ *R* centered around the target. Here, *a* denotes the scale factor between feature layers. The training sample at scale level *n* is *f*_*t*,scale_ (*n*), set to the *d*-dimensional feature descriptor of *I*_*n*_. As shown in Fig. 2b, a 1D Gaussian is implied as the desired correlation output g. The updating of scale filter *h*_*t*,scale_ is like Eq. (6) with the new sample *f*_*t*,scale_.

To estimate the translation of the target, the standard translation filter with raw pixel value and HoG feature are used in DSST [14]. To reduce the computational cost of the DSST tracker, the authors apply PCA-HoG for translation filter learning. Same to the translation filter, compressed scale filter is used without any loss of information (fast DSST, fDSST for briefly). Compared with SAMF method which only uses 7 scale space level, fDSST and DSST use *S* = 33 and *a* = 1.02. This can cover a larger-scale range and more accurate results of scale estimation than in SAMF can be achieved.

Among these three strategies for scale estimation, multi-resolution-based method is simple, but the computing load is higher. Joint scale space estimation is more efficient than the multi-resolution-based method. Iterative joint scale estimation is the most efficient one, though compressive data are used for both the translation and scale estimation, but there is no much information lost.

## The comparison experiments

Though there are few papers which take the scale estimation into consideration in correlation filter-based visual tracking, their results show that the performance of these methods is impressive. It is necessary to make a comprehensive study on the scale estimation issue.

According to the previous section, there are three strategies for scale estimation in correlation filter-based visual tracking. Actually, due to the computational cost of joint scale space estimation, it lost the advantages of the original CF-based method. fDSST is a compressed version of DSST, but there is no much information lost according to their results. Because of this, in the comparison experiments, we just take SAMF and fDSST into consideration.

All experiments are performed on an Intel Duo P8600 2.4 GHz CPU with 8 GB RAM. For the standard DSST method, the default parameter values are *a* = 1.02, *S* = 33, and the standard deviation in the scale dimension of the desired correlation output *g* is set to 1/16 times the number of scales *S*. For the fDSST, the 32-dimensional HOG and intensity combination is reduced to 18 dimensions in our experiments. The dimensionality of the scale features from *d* ≈ 1000 to only *S* = 17 dimensions. The parameter values of SAMF for all videos are set to the same, a Gaussian kernel type and HoG-Color feature type are used, the cell size is 4, and nine orientations are used for HoG. The padding size is set to 1.5.

The methods are quantitatively evaluated under MATLAB 2016b with the datasets of the online tracking benchmark (OTB) dataset [20]. Because we focus on the comparison of scale estimation performance, only the videos marked with scale variation are used. These videos are Biker, BlurBody, BlurCar2, BlurOwl, Board, Box, Boy, Car1, Car24, Car4, CarScale, ClifBar, Couple, Crossing, Dancer, David, Diving, Dog, Dog1, Doll, DragonBaby, Dudek, FleetFace, Freeman1, Freeman3, Freeman4, Girl, Girl2, Gym, Human2, Human3, Human5, Human6, Human7, Human8, Human9, Ironman, Jump, Lemming, Liquor, Matrix, MotorRolling, Panda, RedTeam, Rubik, Shaking, Singer1, Skater, Skater2, Skating1, Skating2.1, Skating2.2, Skiing, Soccer, Surfer, Toy, Trans, Trellis, Twinnings, Vase, Walking, Walking2, Woman. There are totally 57 video sequences used.

Moreover, the tracking results about the three standard evaluation metrics namely overlap precision (OP), distance precision (DP) and tracking speed in frames per second (FPS) are reported in the existing literatures. So we mainly compare the performance on how large-scale variation they can suffer from and can still work under this condition.

According to the ground-truth data, the referent scale value is proposed and can be calculated as follows,

8 $$S_{i} = \frac{{w_{i} *h_{i} }}{{w_{0} *h_{0} }}.$$

Here, the initial scale value *S*_0_ = 1 in first frame, (*w*_0_, *h*_0_) denotes the target width and height in first frame, (*w*_*i*_, *h*_*i*_) is the current width and height, and *S*_*i*_ denotes the current scale value. We have tried several other metric methods, such as the relative width/height change, but the scale value computed by (8) is seemed more practical for the comparison.

Though the video sequences used for the comparison study show a relatively large-scale variation, most of these video sequences also include other attributes. For example, video *Lemming* is included in scale variation (SV), occlusion (OCC), fast motion (FM) and out-of-plane rotation (OPR) [20]. And, moreover, different visual trackers show different performances on these attributes, so the experiment results on these video sequences are not the same. But for most of these videos, both trackers show the consistent scale change trends. Because of this, we only list some typical cases and the causes for the results are analyzed.

**Fig. 3:**
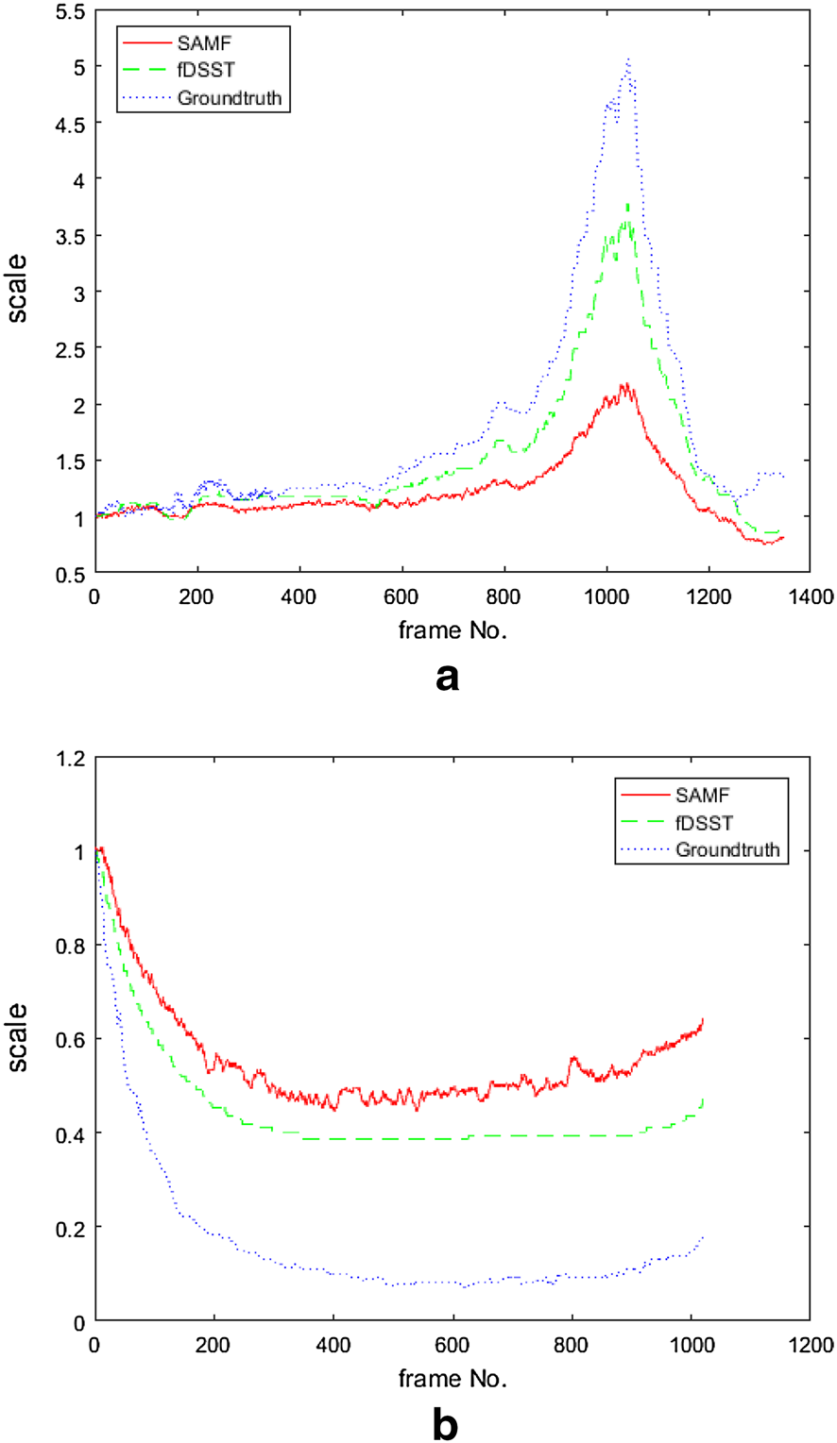
Some videos show the consistent results. **a** Scale estimation results for Dog1. **b** Scale estimation results for Car1

For the consistent results, we select the video Dog1, Car1 as examples, and the experiments show that the scale estimation results of SAMF and fDSST are consistent with the ground truth. As shown in Fig. 3, the blue dotted line is for the ground truth, the green dashed line is the results of fDSST, and the solid red line is the results of SAMF. Although the magnitudes of scale values are not same, it may be caused by the different metric measurement, but the trends of the curve are almost the same.

**Fig. 4:**
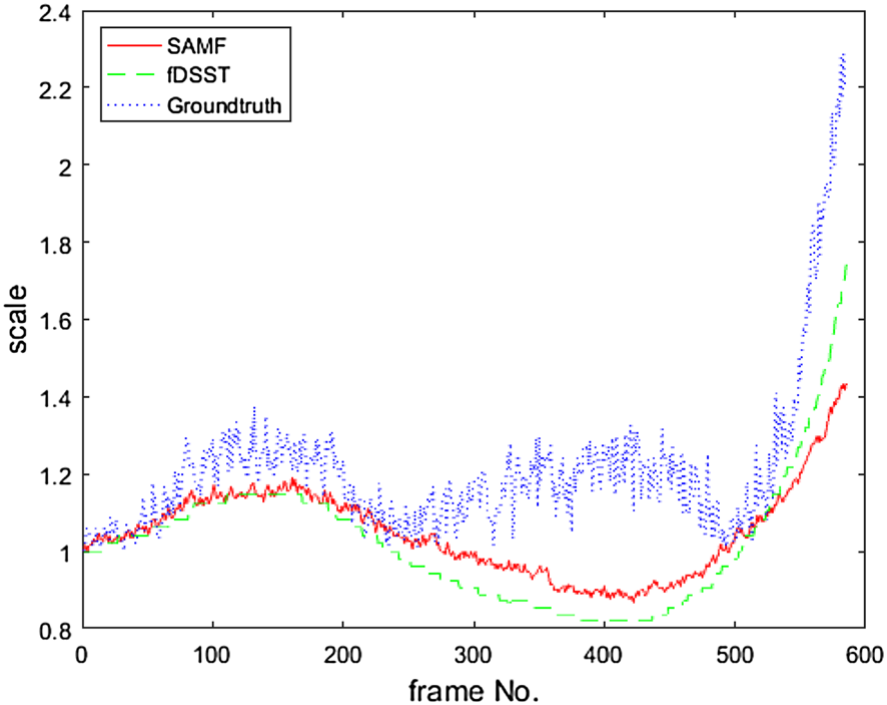
Scale estimation results for BlurCar2

**Fig. 5:**
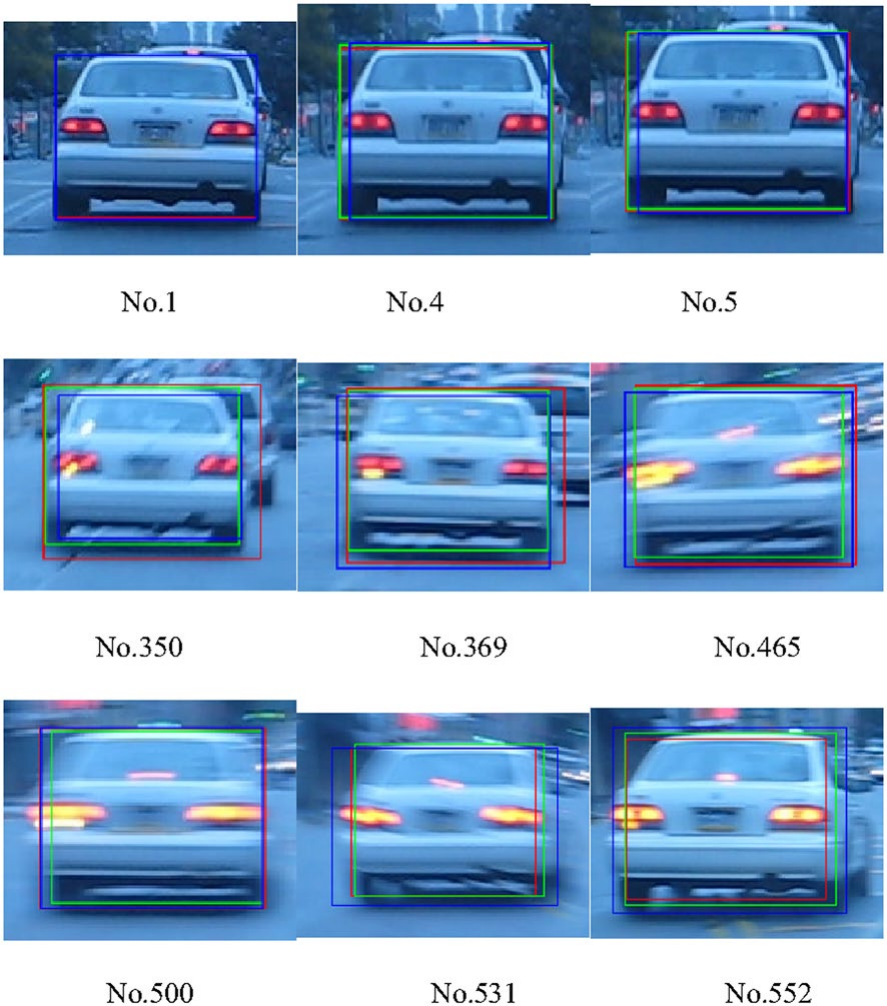
Tracking results of SAMF(R)/fDSST(G)/the reference(B)

The experiment results with videos, such as BlurCar2, as shown in Fig. 4, and the results of SAMF and fDSST show a general resemblance with the ground truth, but small differences in some local sections, for example, from frame number 300–500 in Fig. 4, exist among them. For these figures, we follow the same color label rules; that is, the red color (R) indicates the result of SAMF, the green color (G) presents the output of fDSST, and the blue color (B) represents the ground truth. We pick out some frames from BlurCar2 and show the tracking outputs in Fig. 5. In this experiment, we can see that the ground truth may be not accurate enough, as indicated in frame no. 5. On the other hand, because of the motion blur, it is hard to say which one is more accurate than the others, as indicated in frame no. 369/465/531. In this case, the output of SAM/fDSST and the ground truth are basically same, but there are small differences which may result from inaccuracy of the ground truth.

**Fig. 6:**
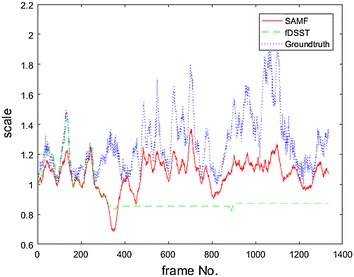
Scale estimation results for Lemming

**Fig. 7:**
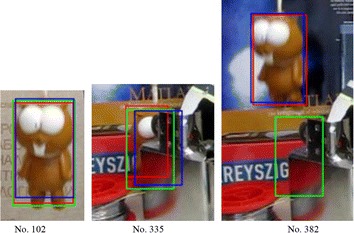
Tracking output of SAMF(R)/fDSST(G)/the reference(B) with Lemming

**Fig. 8:**
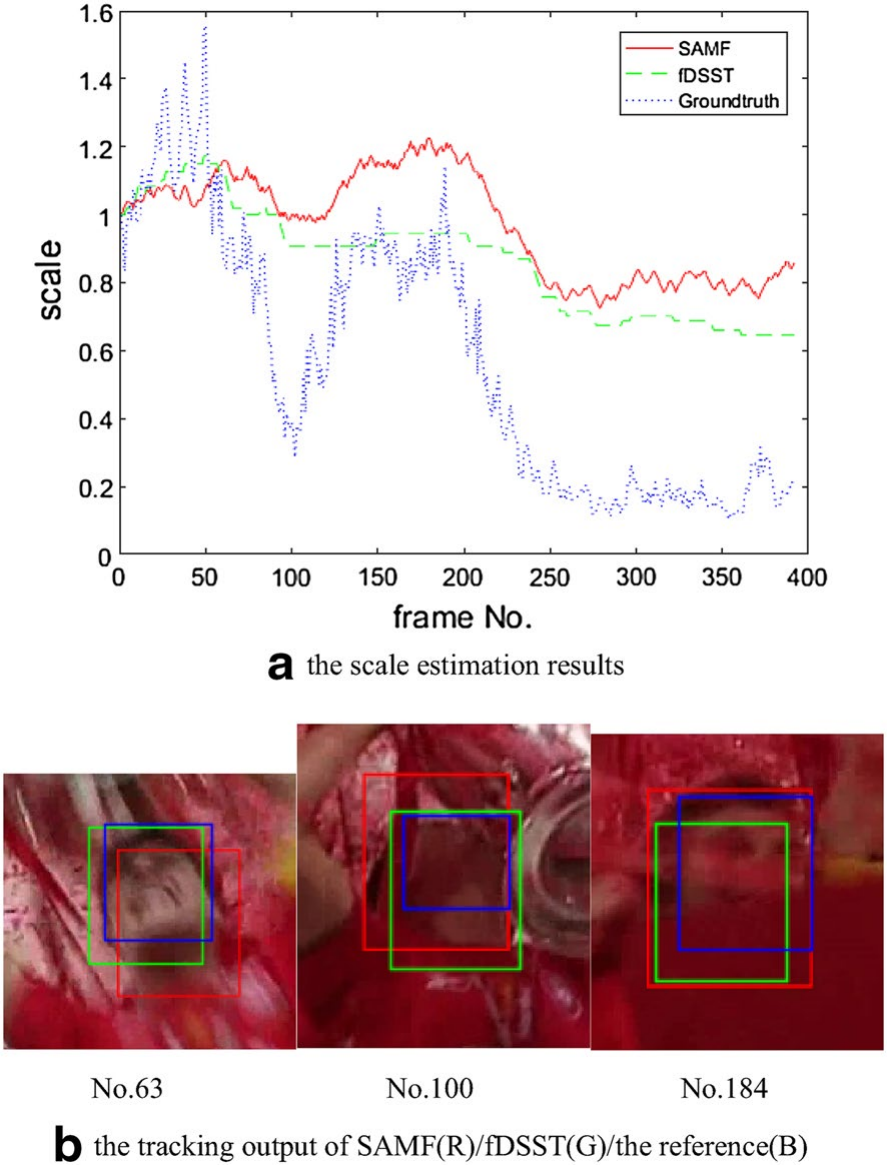
Experiment results with Soccer. **a** Scale estimation results. **b** Tracking output of SAMF(R)/fDSST(G)/the reference(B)

For some videos, such as Lemming and Soccer, the result of SAMF is outperformed over fDSST, as illustrated in Fig. 6. In this experiment, we found that fDSST is failed since frame no. 335, as shown in Fig. 7, because of the occlusion. But SAMF can track the Lemming continuously after the target reappears. It may be benefit from the usage of color-naming feature. Figure 8 shows the results for Soccer, and it can confirm that by making use of color-naming feature, the output of CF-based tracking is more stable.

**Fig. 9:**
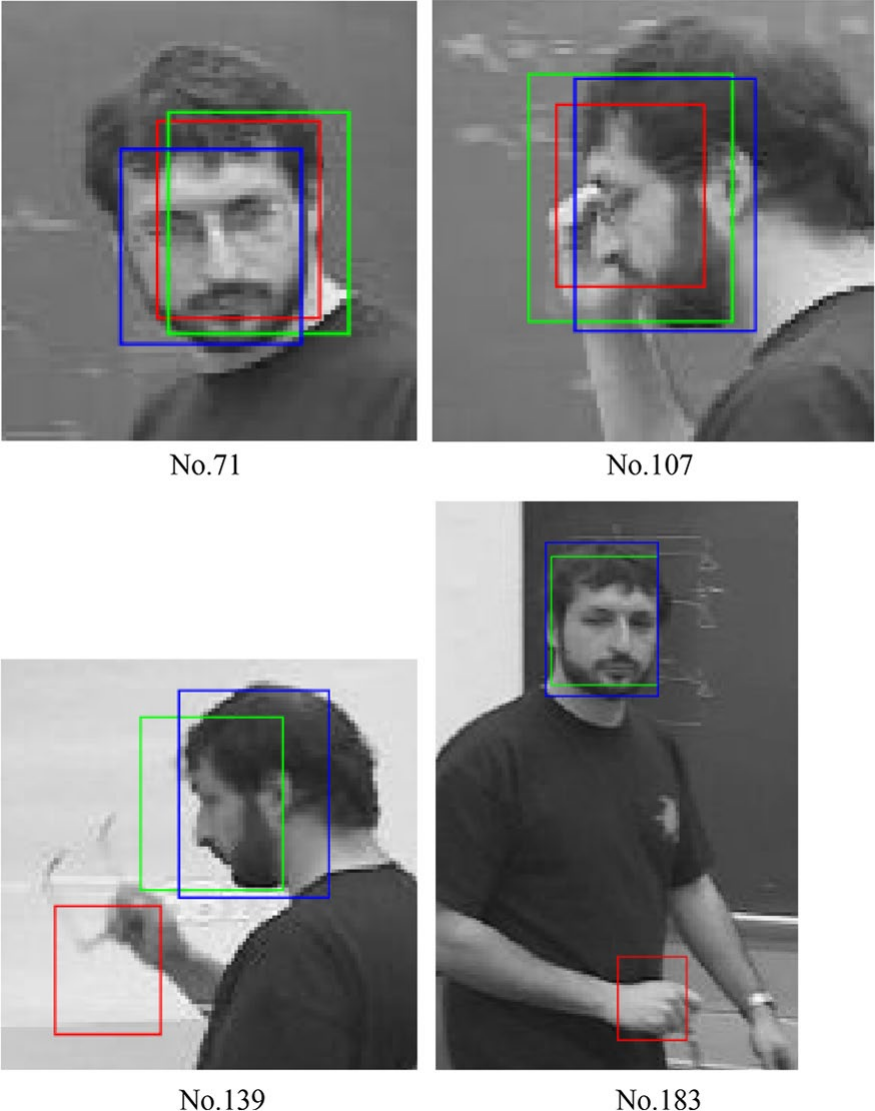
Scale estimation results for Freeman1 (SAMF(R)/fDSST(G)/the reference(B))

For some videos such as Freeman1, the results of fDSST are better than SAMF, as shown in Fig. 9. In this experiment, we found that SAMF is failed after frame no. 139, as shown in Fig. 9b, when Freeman takes off his glass. It is interesting that in this case, however, fDSST can work well. We found that because this video is a gray-level image sequence, this makes the color of the hand and face look like the same. But the color-naming feature is a very important part in SAMF tracker, and the tracker is distracted by hand this time.

## Conclusion and discussions

In this paper, several adaptive scale estimation methods based on correlation filter are analyzed and compared. Both the SAMF and fDSST show their high performance even in some challenging scenarios. The SAMF algorithm integrates the HoG with color-naming feature in a multi-resolution framework, and the computational cost is larger than fDSST, but it shows high performance when the color information is available, such as Soccer and Lemming. It can work more stable and accurate than fDSST and even recover from the occlusion sometime. On the other hand, when only gray-level image is available, the color-naming feature will lose the advantages, because compressive HoG is employed, and fDSST tracker is more efficient. Which tracker is suitable for the application will depend on the efficiency and the accuracy. Both trackers are capable of adaptive scale estimation and greatly improve the tracker’s performance.

 But it is interesting that, for the experiment with Freeman1, the tracker is distracted by the hand color. The reason for this will be further explored. Moreover, how to take the two approaches advantages to cope with the challenging tracking scenarios is the scope of future works.
